# Locating Atrial Fibrillation Rotor and Focal Sources Using Iterative Navigation of Multipole Diagnostic Catheters

**DOI:** 10.1007/s13239-019-00414-5

**Published:** 2019-04-15

**Authors:** Prasanth Ganesan, Elizabeth M. Cherry, David T. Huang, Arkady M. Pertsov, Behnaz Ghoraani

**Affiliations:** 1 Department of Computer and Electrical Engineering, Florida Atlantic University, Boca Raton, FL USA; 2 School of Mathematical Sciences, Rochester Institute of Technology, Rochester, NY USA; 3 Department of Cardiology, University of Rochester Medical Center, Rochester, NY USA; 4 Department of Pharmacology, SUNY Upstate Medical Center, Syracuse, NY USA; 5 777 Glades Road, EE (Bldg. 96) Room 319, Boca Raton, FL 33431 USA

**Keywords:** Atrial fibrillation, Electrogram signal processing, Non-pulmonary-vein source detection, Patient-specific ablation strategy development, AF ablation target detection

## Abstract

**Purpose:**

Multi-polar diagnostic catheters are used to construct the 3D electro-anatomic mapping of the atrium during atrial fibrillation (AF) ablation procedures; however, it remains unclear how to use the electrograms recorded by these catheters to locate AF-driving sites known as focal and rotor source types. The purpose of this study is to present the first algorithm to iteratively navigate a circular multi-polar catheter to locate AF focal and rotor sources without the need to map the entire atria.

**Methods:**

Starting from an initial location, the algorithm, which was blinded to the location and type of the AF source, iteratively advanced a Lasso catheter based on its electrogram characteristics. The algorithm stopped the catheter when it located of an AF source and identified the type. The efficiency of the algorithm is validated using a set of simulated focal and rotor-driven arrhythmias in fibrotic human 2D and 3D atrial tissue.

**Results:**

Our study shows the feasibility of locating AF sources with a success rate of greater than 95.25% within average 7.56 ± 2.28 placements independently of the initial position of the catheter and the source type.

**Conclusions:**

The algorithm could play a critical role in clinical electrophysiology laboratories for mapping patient-specific ablation of AF sources located outside the pulmonary veins and improving the procedure success.

**Electronic supplementary material:**

The online version of this article (10.1007/s13239-019-00414-5) contains supplementary material, which is available to authorized users.

## Introduction

Atrial fibrillation (AF), characterized by an irregular beating of the atria due to disorganized electrical signals, is a major cause of stroke and affects more than 2.7 million people in the US.^3^ Catheter ablation involving isolation of pulmonary veins (PVs) attempts to block the ectopic signals originating from PVs that are believed to be triggering the arrhythmia. Unfortunately, this procedure has a low long-term success rate that is attributed to the formation of additional rotor and focal sources in the atria outside the PV area, especially in patients with more persistent AF.^11^

During AF ablation procedures, the electroanatomic mapping of the left atrium is constructed by maneuvering a multipolar diagnostic catheter along the left atrial endocardial surface. Current non-PV source detection techniques use the single-electrode electrogram characteristics and construct a 3D electro-anatomic AF map of the entire atria and use it to identify non-PV ablation targets.^1^,^6^,^15^,^21^,^25^,^30^ However, the contradictory clinical outcomes from these techniques suggest that there is a need for a new technique that can effectively utilize the information recorded during multipolar diagnostic catheter movements and successfully detect AF ablation targets.^2^,^7^,^31^ Additionally, constructing the 3D electro-anatomic AF map of the entire atria is time-consuming and often requires the acquisition of thousands of local electrograms. One promising approach that is being actively explored is using 64-electrode bi-atrial basket catheters to simultaneously map the entire atria and locate rotor and focal sources in the atria.^22^ The clinical and theoretical studies, however, show that the electrode density that can be achieved using the state-of-the-art catheters is not always sufficient to provide accurate localization and can lead to a significant number of false positives.^4^ As an alternative to basket catheters, the work in Ref. 27 explored multipolar spiral catheters, which provide higher spatial resolution but still need to map the entire atrial surface.

**Figure 1:**
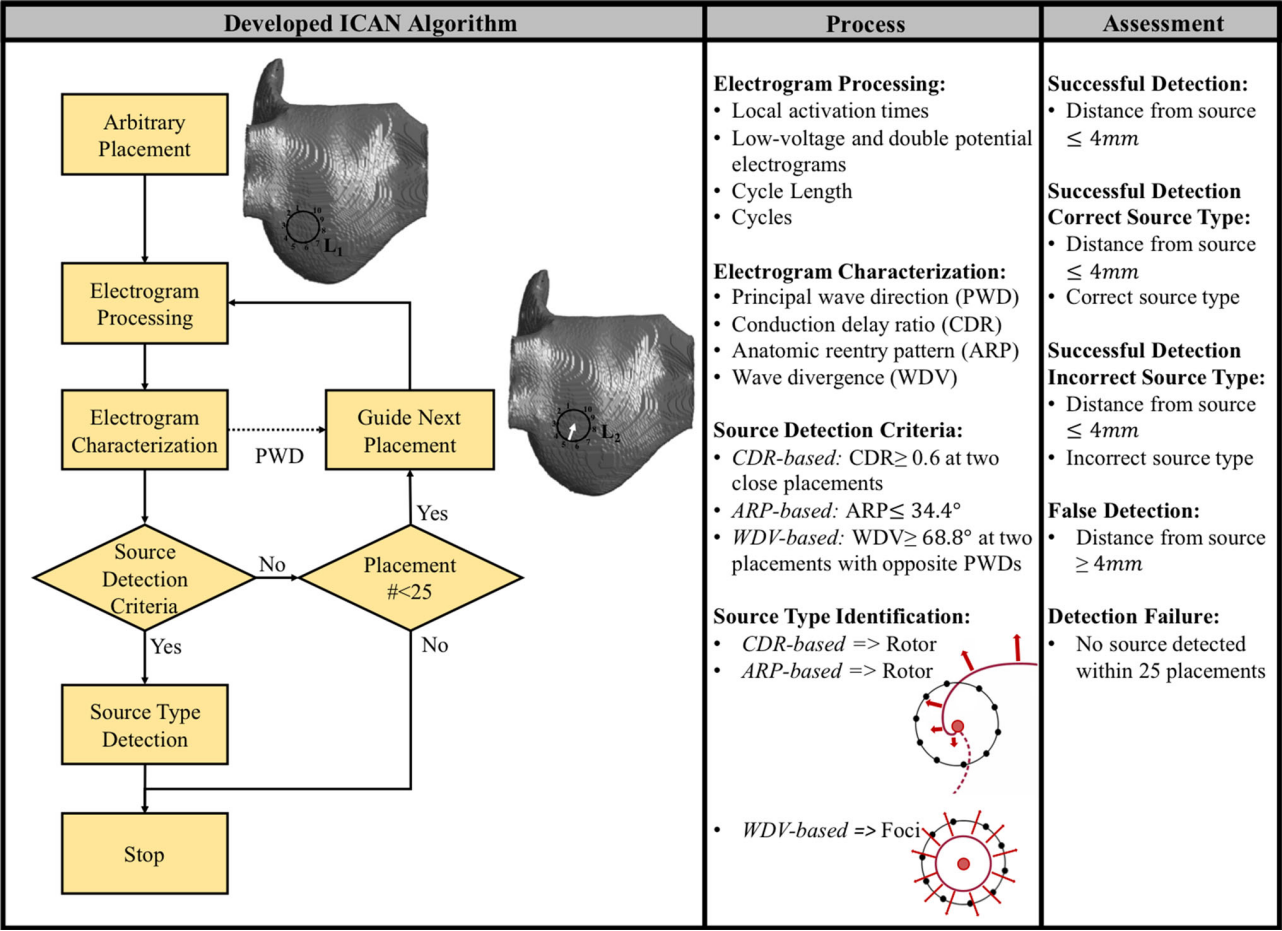
Outline of the proposed iterative catheter guidance (ICAN) and assessments to locate AF sources using a 20-pole, circular diagnostic catheter. The algorithm starts with an arbitrary catheter placement. The electrogram signals are processed and the electrogram patterns are characterized into three source detection criteria to detect whether the current location of the catheter indicates an AF source. If so, the algorithm will stop and report the location and type of the source. Otherwise, the algorithm guides the catheter center to the next location where the process is continued until a source is detected or a maximum iteration of 25 is reached. The located source and detected type is compared against the “ground truth” source trajectory and source type to quantify the ICAN performance into four categories: successful detection (correct source location regardless of the correctness of the identified type), successful detection with correct source type, successful detection with incorrect source type, false detection (incorrect source location), and detection failure (no source was found under 25 catheter placements).

In this paper, we describe a new algorithm, referred to as the iterative catheter navigation (ICAN) algorithm (see Fig. 1), to locate AF sources. Our algorithm is fundamentally different from the existing approaches in three main aspects: (1) instead of using the single-electrode electrogram characteristics, it uses the variations in the electrogram characteristics recorded using a 20-electrode circular catheter; (2) instead of first mapping the entire atria and then locating the AF sources, it iteratively navigates a catheter towards AF sources and does not involve electrophysiological mapping of the entire atrial surface; and (3) it does not make any assumptions on the AF source type to navigate the catheter and as a result, can be used for locating different types of AF sources (rotor and focal sources). We demonstrate that the information obtained from analysis of the activation times derived from the bipolar electrograms is sufficient for the ICAN algorithm to guide the catheter towards the AF source and detect its location and type. The effectiveness of ICAN was tested in the presence of fibrosis and patchy myocardial scars, which makes AF source detection more challenging as is evident by reduced performance of the phase-mapping algorithm due to the low-resolution constraints of a basket catheter.

## Materials and Methods

### Human Atrial Electrophysiology Model

**Figure 2:**
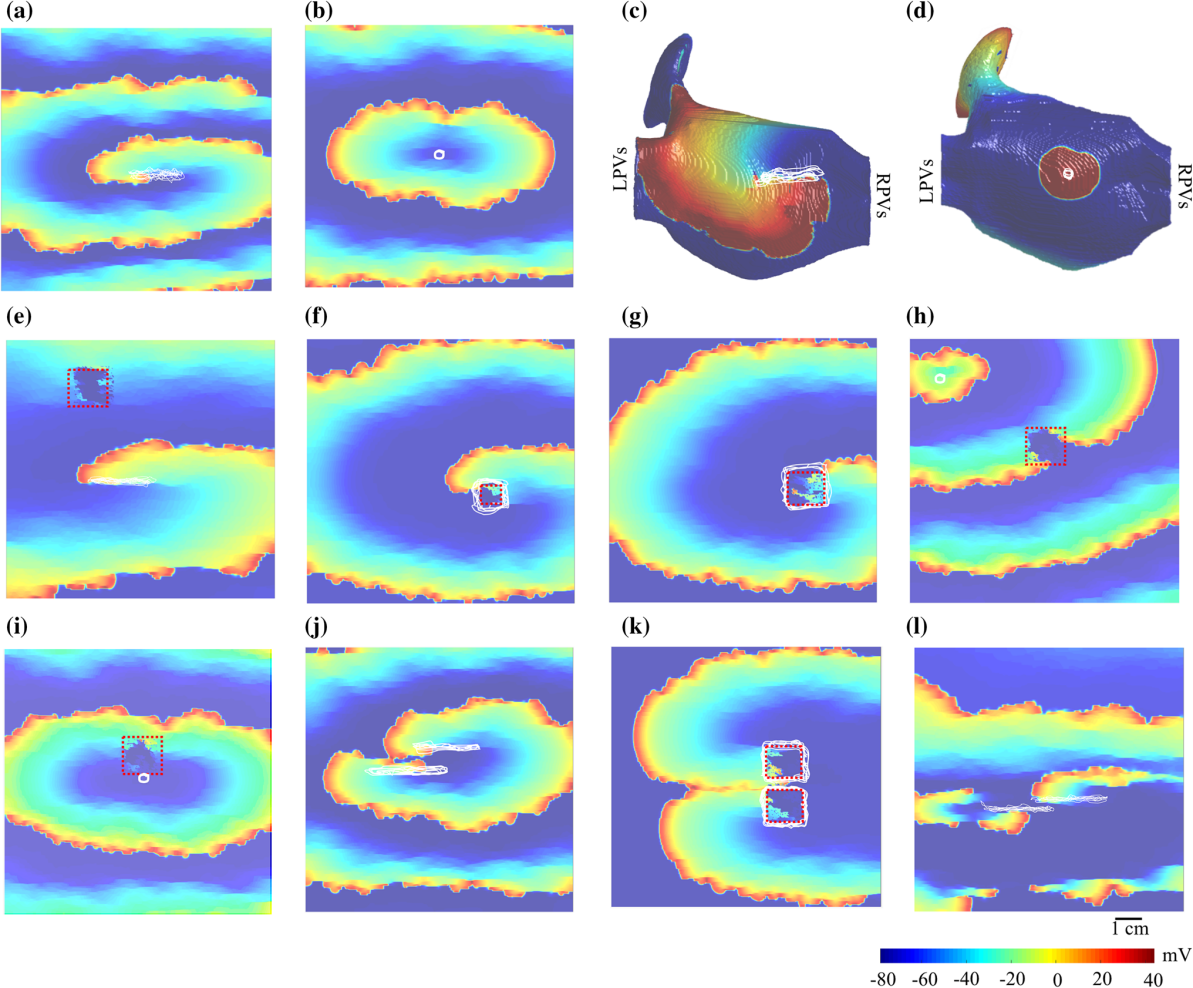
A series of rotor and focal sources are generated. A snapshot of the transmembrane voltage map of 12 of the simulations is shown in this figure. (a) Rotor source, CL = 140 ms. (b) Focal source, CL = 150 ms. (c) Rotor source in 3D geometry, CL = 170 ms. (d) Focal source in 3D geometry, CL = 250 ms. (e) Rotor source with a far myocardial patchy scar with an area of 2.25 cm^2^, CL = 250 ms. (f) Rotor anchored to a small 0.76 cm^2^ myocardial patchy scar, CL = 260 ms. (g) Rotor source anchored to a large 2.25 cm^2^ myocardial patchy scar, CL = 245 ms. (h) Focal source with a far myocardial patchy scar, CL = 150 ms. (i) Focal source close to a large 2.25 cm^2^ myocardial patchy scar, CL = 150 ms. (j) Functional figure-of-eight with two closely placed rotor cores, CL = 180 ms. (k) Anatomical figure-of-eight with a narrow isthmus of 1.25 mm between two 2.25 cm^2^-myocardial patchy scars, CL = 150 ms. (l) Stable fibrillatory cluster of rotors, CL = 170 ms). The white tracings indicate the tip of the source trajectory with longer lifespans. The red dashed lines indicate the myocardial scar region. LPVs: Left Pulmonary veins and RPVs: Right Pulmonary veins.

The simulations were carried out on 2D and 3D human fibrotic atrial tissue based on Nygren *et al*.^23^ ionic model, and time steps of 0.05 ms and the parameters in Ref. 5. We introduced fibrotic changes and ion channel heterogeneity to reproduce complex features of electrograms characteristic of clinical recordings obtained in ablation studies. In some simulations, in addition to fibrosis, we introduced patchy myocardial scars corresponding to “low voltage” areas (Figs. 2e–2i and 2k), which are common for pathological atrial myocardium^12^ as well as fibrillatory activation patterns with non-sustained singularity point rotors^16^ (Fig. 2l). Figure 2 shows 12 of the simulations. See Online Supplements—Section 1 for 2D and 3D AF-simulation details.

### Catheter Simulation

**Figure 3:**
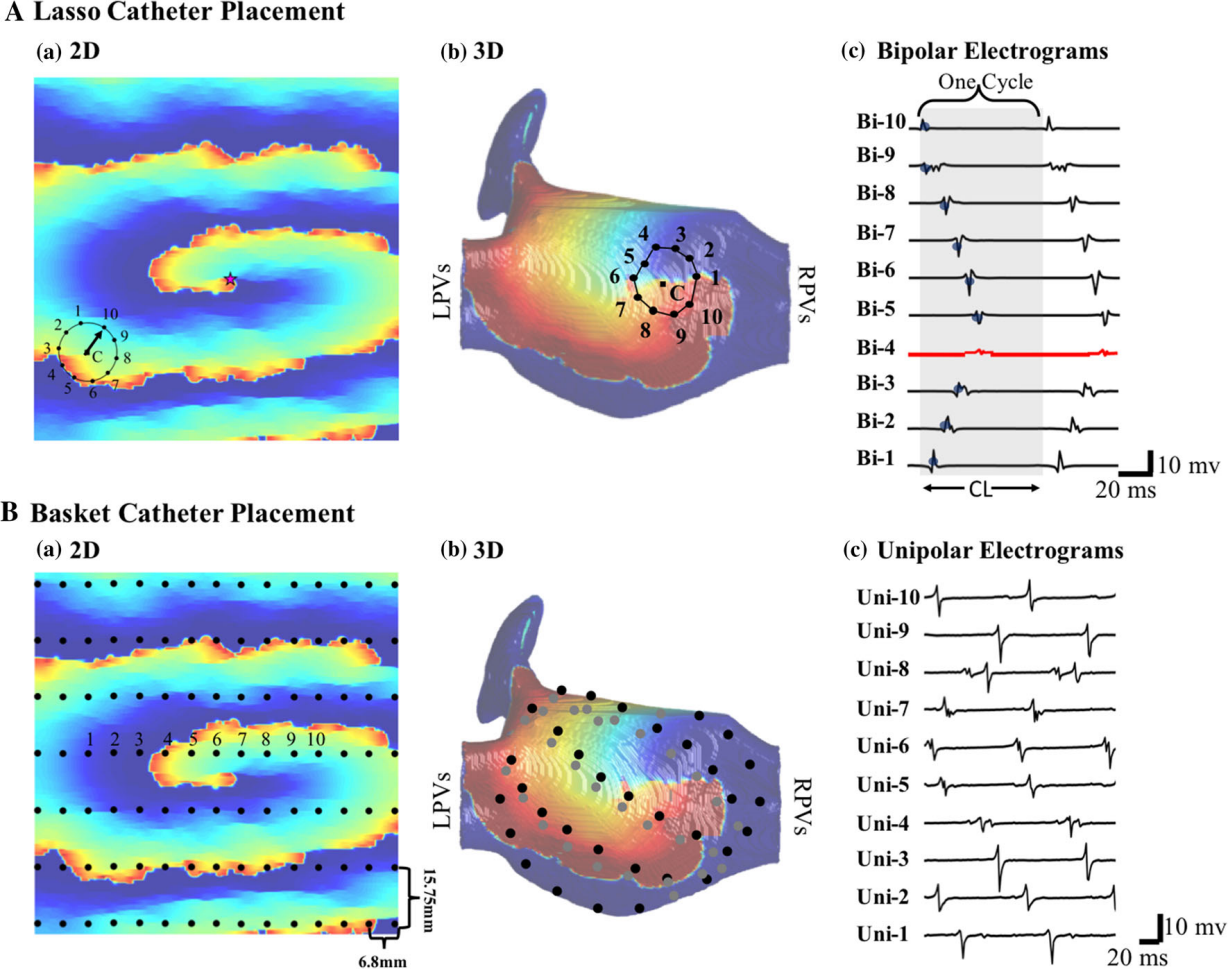
(A) a, b: Simulated 20-pole circular catheter in 2D and 3D atrial geometry. c: A sample bipolar electrograms from the 2D simulation case. The red tracing indicates a low-voltage bipole. The most negative or positive deflections are detected as the local activations (small blue circles). The gray box shows one wave-propagation cycle. The black arrow in figure a shows the PWD as determined by the direction of earliest activated bipole (Bi-10 in this example) with respect to the Lasso center. (B) a, b: Simulated 64-electrode FirMap (Abbott Electrophysiology) atrial basket catheter with diameter 40 mm (the highest resolution) in 2D and 3D atrial geometry. The gray electrodes in the 3D simulation shows the electrodes on the anterior side. c: The simulated unipolar electrograms in unipoles 1 to 10 from the 2D simulation. The simulations correspond to simulation cases A (for 2D) and C (for 3D) from Fig. 2.

We simulated a 20-electrode Lasso catheter (Biosense Webster), with a 7.5 mm radius and 4.5–1–4.5 mm inter-electrode spacing on both 2D and 3D atrial tissue (Fig. 3). In 2D atrial tissue, the catheter was simulated by placing 20 electrodes in a circular pattern according to Eq. (1).

1 $$u_{1} \left( n \right) = \frac{r}{\Delta h}\left[ {\begin{array}{*{20}c} {\cos \left( {\theta_{u} n + \theta_{b} + \phi } \right)} \\ {\sin \left( {\theta_{u} n + \theta_{b} + \phi } \right)} \\ \end{array} } \right] + \left[ {\begin{array}{*{20}c} {x_{c} } \\ {y_{c} } \\ \end{array} } \right]\quad {\text{and}}\quad u_{2} \left( n \right) = \frac{r}{\Delta h}\left[ {\begin{array}{*{20}c} {\cos \left( {\theta_{u} n - \theta_{b} + \phi } \right)} \\ {\sin \left( {\theta_{u} n - \theta_{b} + \phi } \right)} \\ \end{array} } \right] + \left[ {\begin{array}{*{20}c} {x_{c} } \\ {y_{c} } \\ \end{array} } \right]$$

where $u_{1} \left( n \right)$ and $u_{2} \left( n \right)$ indicate the coordinates of $n = \{ 1, \ldots ,10\}$ unipolar pair for a catheter centered at $(x_{c} ,y_{c} )$, $r = 7.5$ mm is the catheter radius, $\Delta h = 0.25 {\text{mm}}$ is the spatial resolution, $\theta_{\text{u}} = 34.4^\circ$ is the spacing between unipoles, $\theta_{\text{b}} = 3.8^\circ$ is half of the distance between a unipolar pair, and $\phi \in \left[ {0,360^\circ } \right]$ is a random value to account for catheter rotation. To simulate a catheter on the real 3D anatomy, the plane of principal curvature was determined using eigenvectors of catheter vertices, from where the normal vectors were projected and registered to the surface using 3D geodesic distance.^28^

In order to compare the performance of the ICAN algorithm with the phase-mapping approach, we simulated a 64-electrode FirMap atrial basket catheter. A total of 120 2D-catheter placements and three 3D-catheter placements was generated by shifting and rotating the basket catheter on the atrial tissue. See Online Supplements—Section 2 for basket catheter simulation shown in Fig. 3B.

### Simulation and Processing of Electrograms

The unipolar electrograms were calculated with a sampling frequency of 500 Hz for each electrode using Eq. (1) in Ref. 10. Bipolar electrograms were calculated as a voltage difference between adjacent unipolar recordings. An example of simulated electrograms is shown in Fig. 3.

At every catheter placement, 1 s recordings were analyzed. Local activation times of each bipolar electrogram were identified as the maximum peak positive or negative deflection within a refractory period of 50 ms. The bipoles with two negative deflections separated by 10 to 50 ms were identified as *long double potentials.*^17^ Cycle length (CL) was determined as the median of the time delays between two consecutive local activations at every bipolar electrogram. Electrogram voltage was defined as the peak-to-peak voltage and bipoles with voltages less than 0.1 mV were defined as *low*-*voltage.*^14^ The activation times associated with the same wavefront were identified as one *cycle* (Fig. 3A) using the calculated activation times and CL. Starting from one of the activation times, the algorithm finds the closest activation times in the adjacent bipoles (within half of the CL and skipping the low-voltage bipoles) until all the activation times of all the bipoles are associated together. This process is repeated for the rest of the recording until all the cycles within 1 s of the recording are identified. The earliest activated bipole within each cycle (bipole 10 in Fig. 3A) is also determined for further analysis.

### ICAN AF Source Detection Using a Circular Catheter

Our ICAN algorithm guides the incremental movements of a circular catheter from an arbitrary initial placement on the atrial tissue until a source of arrhythmia (rotor or focal) is detected by the algorithm (Fig. 1). To do so, we determine principal wave direction (PWD), conduction delay ratio (CDR), anatomic re-entry pattern (ARP), and wave divergence (WDV) at every catheter placement using the identified cycles and their corresponding activation times along with the catheter-guidance trajectory.

The PWD is estimated as the direction to the propagating AF wave source relative to the catheter’s current location. A cycle’s PWD (black arrow in Fig. 3A), is determined as a vector starting from the catheter center and pointing to the earliest activated bipole. The PWD of a 1 s electrogram recording is then determined as a vector with an angle equal to the average PWD angles of all the cycles within the recording in reference to the catheter center.

The CDR is calculated as the average ratio of the total conduction delay to CL for all the identified cycles within the 1 s electrogram recording. For every cycle, the total conduction delay is determined by summing the time differences between the local activations of a bipole and its following counter-clockwise bipole. The process starts from the earliest activated bipole while skipping over any low-voltage bipoles and excluding the bipole preceding to the earliest activated bipole. In the case of double potentials, the shorter delay is selected as the conduction delay.

To calculate ARP, first the average location of *N* consecutive catheter placements, before the current placement, with low-voltage or long-double-potential bipole electrograms is calculated. Then, the ARP is calculated as $360^\circ - 34.4^\circ \times \sum\nolimits_{n = 1}^{N} {e_{n} }$ where $e_{n}$ is the number of low-voltage or long-double-potential bipole electrograms that fall between − 180° and + 180° from the intersect of the catheter circle with a line of average location and the catheter center at *n*th catheter placement. In this calculation, 34.4° is the angle between two adjacent bipoles.

The WDV is computed as the standard deviation of the AF propagation at a catheter placement. The 10 bipoles are grouped into triangular electrodes. For every cycle, linear isochrones are determined using the activation times of the three bipole electrograms. The normal vector from the earliest activation bipole location that is perpendicular to the isochrones is determined as the wave direction. The standard deviation (smallest angle < 180°) of the normal vectors from all the possible triangular electrodes determines the WDV.

#### AF Source Detection Criteria

A source is considered detected if the algorithm detects one of the following three criteria where the first two identify a rotor and the third one identifies a focal source detected:

*CDR-Based Criterion for Rotor* when two close catheter placements (within 1 cm) have a CDR ≥ 0.6, the average location of the two placements is detected as a rotor core. This criterion will be satisfied when the catheter is placed over or near the center of the rotor meandering path and an almost perfect circulatory excitation along the perimeter of the catheter. We avoid the detection of isolated and non-rotor relevant catheter placements with CDR ≥ 0.6 as rotors by enforcing the criterion to be satisfied only when two close catheter placements (within 1 cm) satisfy such a condition.

*ARP*-*Based Criterion for Rotor* when the calculated ARP is ≤ 34.4° (the angle between adjacent bipoles), the average location of the catheter placements that satisfied that ARP criterion is determined as a rotor core. This criterion is satisfied where a rotor core is anchored to anatomical barriers in the form of a patchy myocardial scar or fibrosis, and the catheter is guided to follow a rotor meandering path circling an anatomical barrier.^9^,^12^

*WDV*-*Based Criterion for Foci* when a catheter placement has WDV ≥ 68.8° (2 × 34.4°) and the smallest angle between the PWD vectors at this location and a close catheter placement (within 1 cm) is more than 103.2° (4 × 34.4°), the center of the catheter is detected as a focal source. This criterion will be satisfied when the catheter encompasses a focal source with waves propagating from the inner core of the catheter. False detections at isolated locations are avoided by ensuring that two of such catheter placements are recorded: one when the focal source is inside the catheter and one with the source being outside, which is confirmed by an almost opposite WDV (≥ 103.2°) between the two placements.

#### Determining the Next Location of the Catheter

If the rotor is not detected, the algorithm shifts the catheter center by 7.5 mm (catheter radius) in the direction of the PWD, which defines the direction to the propagating AF wave source relative to the catheter’s current location. At the new placement, the algorithm checks the AF source detection criteria for the presence of a rotor or focal source. This process is repeated until a source is identified or a maximum number of placements (25 in this study) is reached.

#### Constructing Voltage Map

For visualization purposes, an electrogram voltage map is constructed using natural neighbor interpolation technique^29^ as the catheter is navigated on the tissue.

### Detecting AF Sources Using Basket Catheter

We implemented a phase-mapping method to detect rotors^18^ and velocity-of-divergence mapping^26^ to detect focal sources using a 64-electrode FirMap atrial basket catheter. Online Supplements—Section 3 describes the methods in details.

### Quantifying AF Source Detection

For every guidance, the ICAN algorithm provides the location and type of the detected source. The location of the detected source is considered correct if it is within 4 mm (an ablation catheter tip diameter) from the average trajectory of the source. A test is considered a *successful detection, successful detection with correct source type, successful detection with incorrect source type, false detection,* and *detection failure* as described in Fig. 1.

For AF detection using a basket catheter, the algorithms provide the location and type of all the detected sources for every simulation case. A detected source is considered a *true positive* if its location is correct; otherwise, it is considered as a *false positive*. The ratio of the number of true positives to the total number of detected sources is calculated as the *successful detection rate* every time the algorithms are applied to a simulation case with a specific basket catheter orientation.

## Results

### Validation of ICAN in Detecting AF Sources

**Figure 4:**
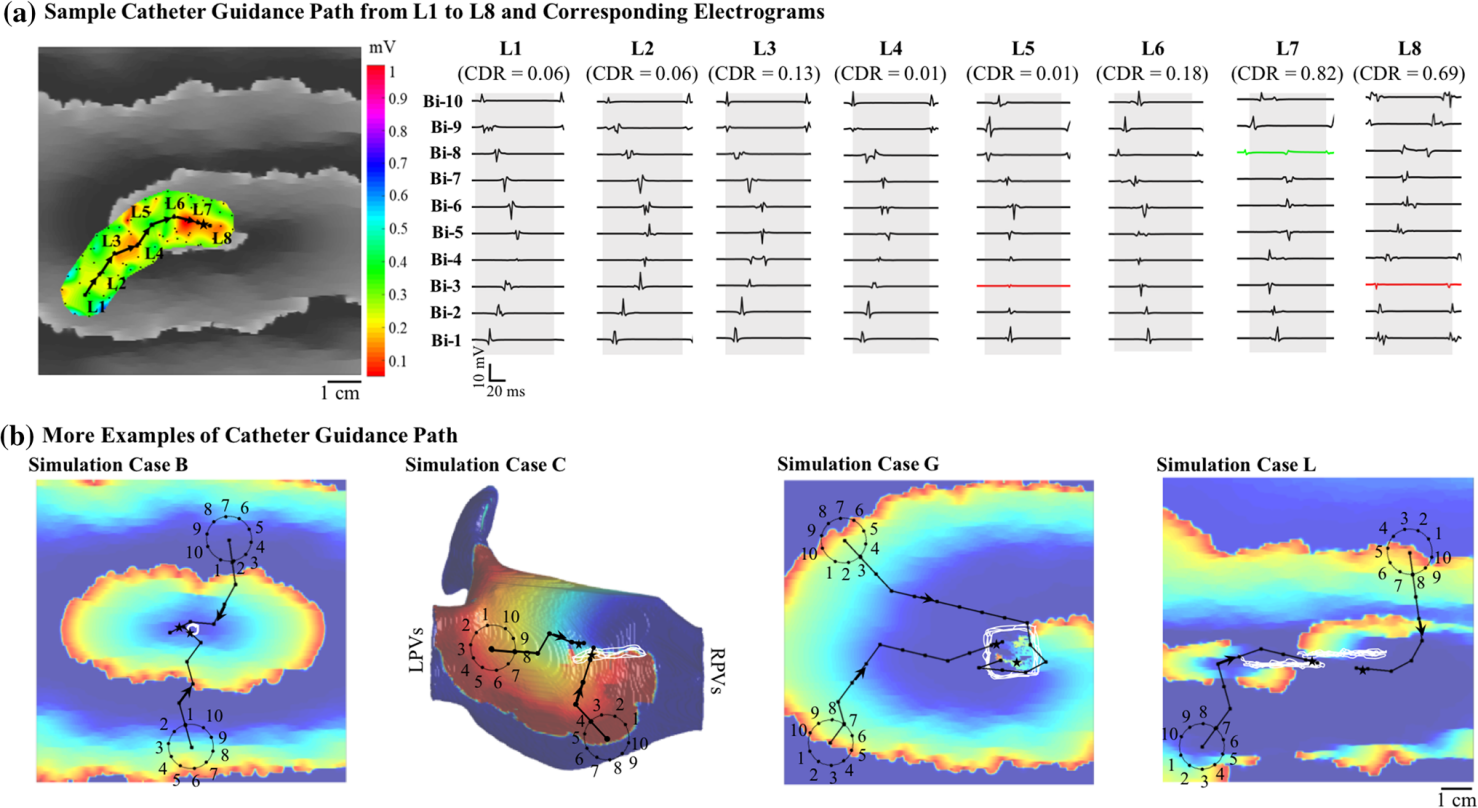
(a) An example of catheter guidance and source localization from an initial catheter location L1 to L8 and one cycle electrograms from every corresponding catheter placement. The CDR values at L6 and L7 (0.73 and 0.78, respectively), were both greater than threshold (*th *= 0.6), thus meeting the CDR-based criterion. The algorithm placed the rotor between the L6 and L7 locations. Here, simulation case A from Fig. 2 is used. The electrogram voltage map as the catheter is guided is constructed and shown (color map). The voltage map is constructed by interpolating the peak-to-peak voltage of the bipolar electrograms on an interpolation grid of 0.25 mm spacing and interpolation area with a radius of 15 mm (diameter of the catheter). (b) Catheter guidance paths from two different locations are shown for four simulation cases from Fig. 2. The black arrows show the iterative guidance path. The located source is shown by a black asterisk.

Our first task was to test algorithm convergence from different initial conditions. A catheter path to the rotor and the recorded electrogram at each catheter placement are displayed in Fig. 4a. Starting from an arbitrary placement (L1), the algorithm guided the catheter to the subsequent placement at L2 until it reached L7 placement, where the ICAN algorithm identified the rotor. Figure 4b shows two more catheter guidance examples on four different simulations.

**Figure 5:**
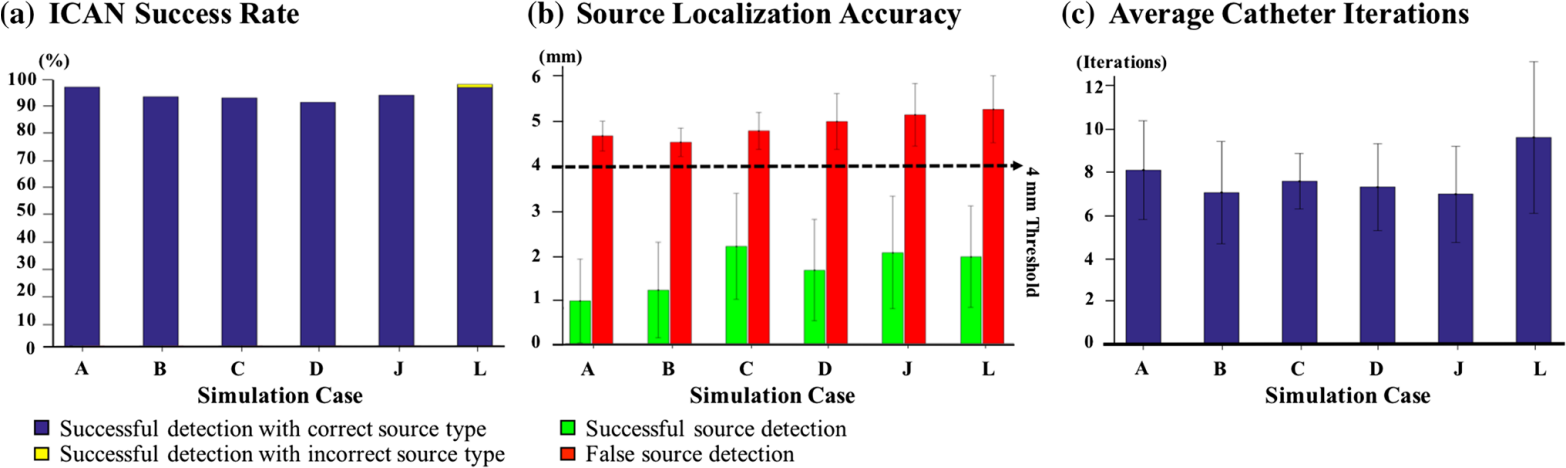
Starting from 114,921 uniformly spaced initial catheter locations across the simulated region, ICAN guided the subsequent placements of a Lasso catheter until it detected the location and type of an AF source or a maximum catheter placement of 25 was reached. (a) Percentage of successful source detection. The successful source detections with correct and incorrect source types are shown in different colors. (b) The average distance of the detected source with the “ground truth” source trajectory. The successful and false detections are shown in two different colors. (c) The average number of catheter placements until a source was detected. None of the trials exceeded 25 catheter placements. The simulation cases A–D, J, and L are as shown in Fig. 2.

To further test the efficacy of our algorithm, we placed the catheter on 114,921 initial catheter locations across the simulated region for different rotor and foci mechanisms. The average rate of successful detection for a rotor and focal 2D and 3D simulation case, as well as functional figure-of-eight re-entry and fibrillatory cluster of rotors are shown in Fig. 5a. The average rate of successful detection was higher than 92.02% in all six cases. The source type was correctly identified in all cases with a successful detection (blue in Fig. 5a) except for one case where 1.05% of the successful detection was with incorrect source type (yellow in Fig. 5a).

Figure 5a shows the average distance of the located sources from the ground-truth source trajectory. Note that the average distance of the false detected rotors was only 4.91 ± 0.52 mm away from the ground truth (red bars in Fig. 5b), which indicated that the false detected sources were not too far from the rotor meandering path. No detection failures were reported, and always an AF source was located before 25 catheter placements.

Figure 5c shows the average number of catheter placements until the source is detected. On average, a total of 7.56 ± 2.28 iteration took for ICAN to guide the catheter towards the source over all the six cases. As expected, the number of catheter placements required to find the source correlated with the distance between the initial placement of the catheter and the source. Our analysis showed that the number of placements before a source was detected linearly increased with a rate of 6.09 mm per iteration as a function of the distance of the initial catheter placement. This suggests that ICAN efficiently navigates the catheter towards the source compared to a direct path to the source with a rate of 7.5 mm (i.e., the catheter radius and guidance steps).

### Robustness to CL Change and Poor Catheter Contact

**Figure 6:**
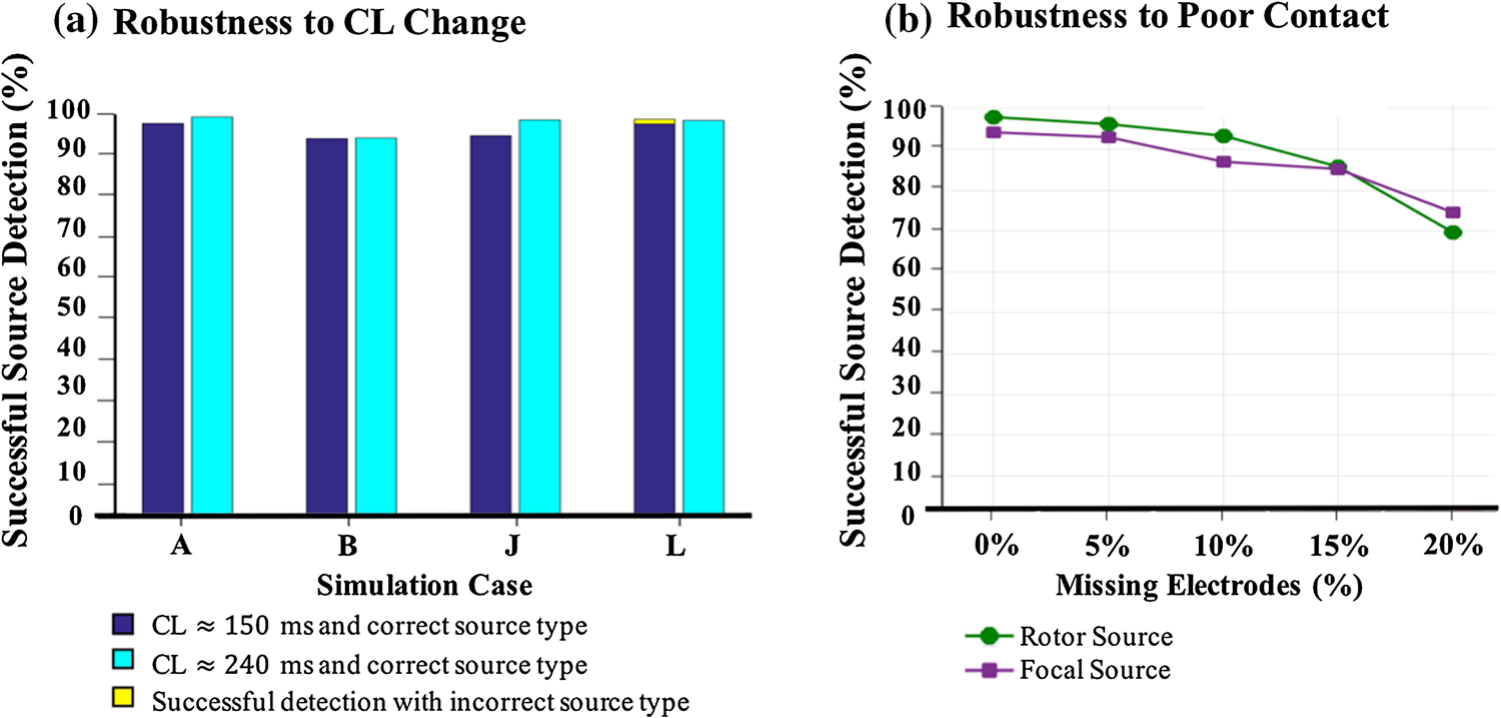
(a) The percentages of successful source detection using ICAN for two different CL values of approximately 150 and 240 ms are shown by the bars with two different colors. The simulation cases A, B, J, and L are as shown in Fig. 2. (b) The change in the percentage of successful source detection of ICAN with a missing electrode rate of 0 to 20% for a rotor source (simulation case A in Fig. 2) and a focal source (simulation case B in Fig. 2).

The robustness of ICAN to change in arrhythmia cycle length is evaluated, and the results are reported in Fig. 6a. With the increase of CL, we observed a slight increase in the source detection accuracy and source type detection in case of a rotor (Fig. 2a), figure-of-eight re-entry (Fig. 2e), and fibrillatory cluster of rotors (Fig. 2f) simulations, and no change in case of focal simulation (Fig. 2b). The reason for the increase could be due to the improvements in the cycle detection algorithm (“Simulation and Processing of Electrograms”) when there is a larger gap between cycles as the CL of the arrhythmia increases.

We evaluated the efficacy of ICAN for source detection with respect to poor catheter contact with the endocardium. In order to simulate the effect of missing electrodes due to poor contact, at every catheter placement, $n \in \{ 1,2\}$ electrodes were randomly selected and removed from the electrogram processing. The ICAN algorithm guided the catheter and detected the location and type of the AF source without the electrograms of the missing electrodes, and the results are shown in Fig. 6b. The percentage of missing electrodes was calculated as the ratio of the missing electrodes to the total electrodes (rounded to the nearest five) for every catheter guidance path. As expected, poor contact reduced the performance; however, it is notable that ICAN was able to detect a source with an average success rate of greater than 85% even with 15% missing electrodes.

### Performance of ICAN with Patchy Myocardial Scars

**Figure 7:**
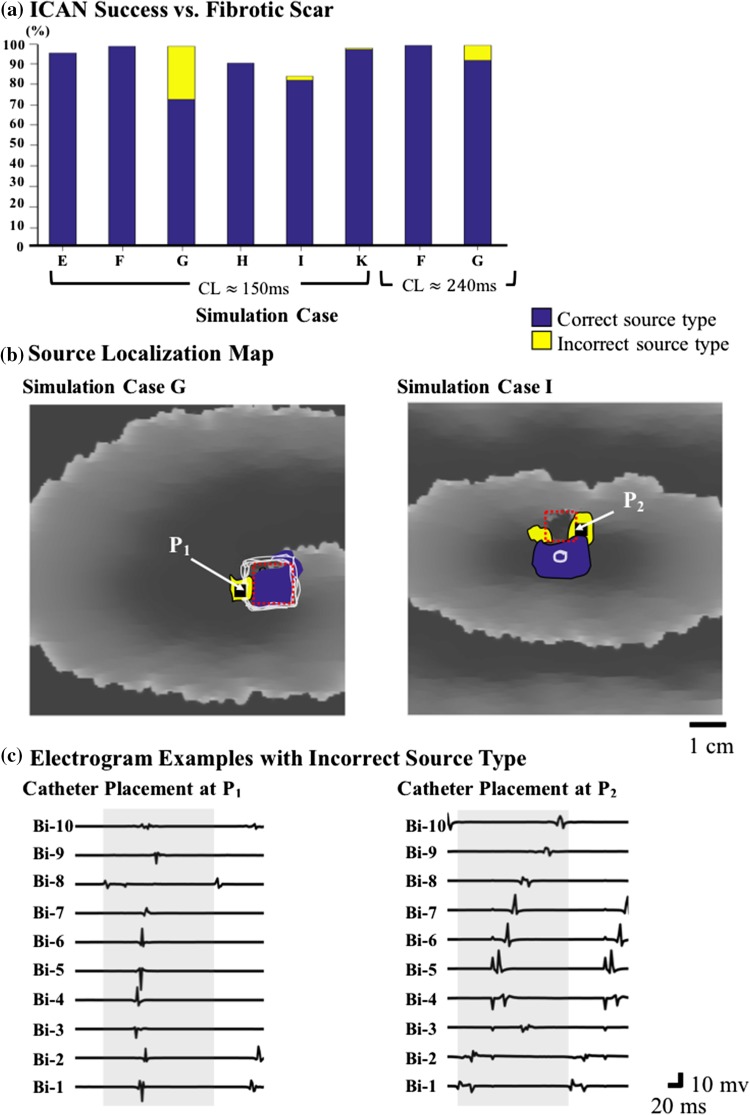
(a) The percentage of successful source detection with the ICAN algorithm for different simulation cases and the presence of patchy myocardial scar. (b) The localized sources with a catheter starting from a total of 114,921 uniformly spaced initial positions are shown for two simulation cases. Different colors represent located sources with correct and incorrect source types. (c) The bipole electrograms of two located sources with an incorrect source type are shown. The catheter placement P_1_ is a rotor that is identified as a focal source and vice versa for the catheter placement P_2_. Simulation cases are as shown in Fig. 2. The gray box in (c) is as described in Fig. 3A.

Our testing was carried out in the presence of patchy myocardial scars corresponding to “low-voltage” areas, which makes source detection more challenging. As shown in Fig. 7a case E, a far myocardial scar has a negligible effect on the rotor detection (96.20% vs. 97.90 with no patch scar), and no source was detected around the scar. The ICAN detection rate was increased when the rotor anchored to a low voltage zone (cases F and G). In these particular cases, the wave circulated a large unexcitable patch producing a large anatomical barrier. In cases like this, ICAN establishes a guidance path around the unexcitable patch and assigns the center of the path as the rotor location using the ARP-based criterion (see Fig. 4d as an example). Using both rotor detection criteria, ICAN was able to detect rotor sources with a 99% detection rate regardless of the scar size. An interesting observation was that the percentage of the rotor sources that were detected as a foci increased with the size of low-voltage area (as shown by yellow color in Fig. 7a, case G). All the localized sources (both successful and false) for simulation case G is shown in Fig. 7b where the yellow area indicates the catheter guidance paths that resulted in incorrect detected source types. The electrograms of a cycle at one of the locations (P_1_) that was identified as a focal source is shown in Fig. 7c. As shown, there is a rapid propagation along the Lasso bipoles that appears as a focal source with simultaneous activation. This effect, however, decreased as the CL was increased from 150 to 240 ms (the yellow area of case G with two different CL values Fig. 7a) and also in case of anatomical figure-of-eight re-entry with a narrow isthmus between two patchy myocardial scars (case K).

In the case of the focal source (Fig. 7a, case H and I), there is a slight decrease in the ICAN detection rate. The reason is the slow conduction at some locations around the low-voltage area appears as a rotor (see electrogram sample in Fig. 7c, P_2_). Hence, depending on the starting location of the ICAN algorithm, some of those locations were detected as a rotor source (yellow areas in Fig. 7b, case I). Since some of those detected sources were far from the focal source, there was a slight decrease in the success rate of ICAN for detecting the focal source in the presence of a low-voltage scar area.

### Detecting AF Sources Using Basket Catheter

**Figure 8:**
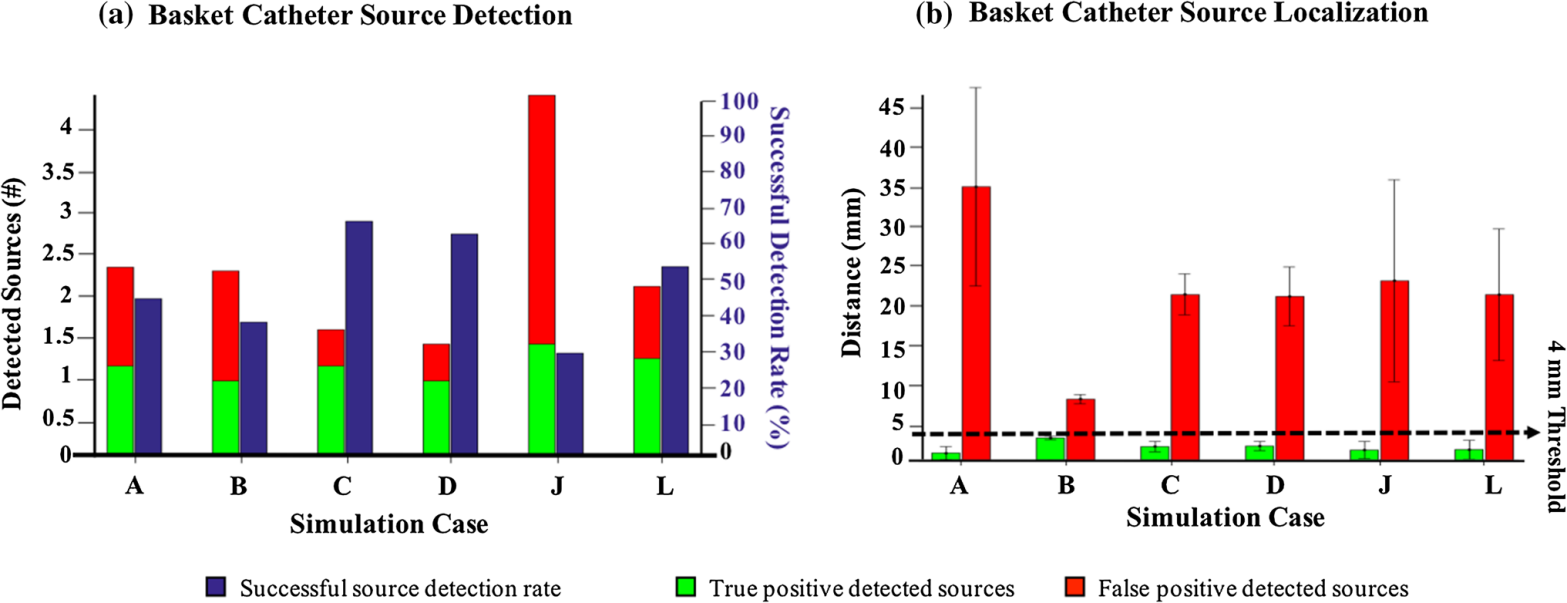
(a) The average number of detected sources using the 64-electrode basket catheter and the algorithms described in “Detecting AF Sources Using Basket Catheter” for different catheter placements (see “Catheter Simulation”). The average number of true and false positives are shown in different colors. The percentage of the true positives to the detected sources is calculated as the successful source detection rate and its average over all the catheter placements is shown in a separate bar with the right-hand side scale. (b) The average distance of the detected sources to the ground truth source for the true positive and false positive detected sources are shown in bars with different colors. Simulation cases are as shown in Fig. 2.

The phase-mapping and velocity-of-divergence mapping techniques were applied to the unipolar electrograms that were collected using a basket catheter. For every simulation case, a set of catheter placements were used (120 in 2D and three in 3D simulation cases). The average number of detected sources and true and false positives is shown in Fig. 8a. In most cases, the algorithms detected more than one source resulting in an average successful detection of less than 50% in all cases expect the 3D rotor and focal source and fibrillatory cluster of rotors (Fig. 8a, cases C, D, and L). This is while ICAN provided consistent performance for all cases with a significant difference, *p* value < 10^−18^. In addition, the located false rotors were far (21.32 mm ± 8.51 mm) from the rotor meandering path (Fig. 8b), while the ICAN method was accurate, with the average error exceeding 5 mm in very few cases (Fig. 5b). The average distances of the false detected rotors for the phase-mapping method were higher than 20 mm for all the simulation cases expect for a focal source. We did not find any case in which no source was detected at all.

## Discussion

A novel iterative catheter navigation algorithm was presented that locates a rotor or focal source using a conventional 20-electrode catheter without the need for full mapping of the entire atria in advance or any assumption of the source type. ICAN guides a catheter from an arbitrary initial placement on the atrial endocardial surface towards the location of an AF source and identifies the focal or rotor type. ICAN was tested on realistic simulated data with several test cases of a rotor and foci-driven arrhythmias (see Fig. 2), which covered a broad range of activation patterns in 2D as well as 3D AF simulations. In all cases, we modeled globally distributed fibrosis.

### Efficiency of ICAN in Detecting AF Sources

Our analysis showed that our algorithm is capable of identifying not only stable and unstable rotors but also focal sources as well as figure-of-eight re-entry. The high performance of the algorithm was evident by high source detection success rate (Fig. 5a). Also, the false detected sources with ICAN were not too far from the source and formed a dense cloud of points in the vicinity of the source (Fig. 5b). It should be noted, however, that robustness and high resolution mean an iterative process must occur. On average, it takes 7-12 catheter placements to localize the source, and more or fewer placements may be needed depending on the initial distance of the catheter from the source (Fig. 5c). There was no significant difference between the method’s performance in 2D and 3D simulations with realistic geometry. This observation was expected as these cases do not offer fundamentally different scenarios that could confound our algorithm. As long as we can accurately derive the local activation times, the algorithm will guide the catheter to a source on the atrial surface no matter how complex the surface geometry and the pattern of wave propagation. Furthermore, ICAN’s ability to detect both rotor and focal sources indicates that it is also able to detect 3D scroll waves.^24^ 3D scroll waves can be divided into two distinct groups with filaments intersecting the myocardial surface and the ones that do not intersect with the surface. In the case of the former, the scroll wave filament manifests itself as a rotor, and in case of the latter, it will appear as a breakthrough or a focal activation pattern. Focal sources can be produced by other types of intramural re-entry as well,^8^,^13^ which suggests that the ICAN source detection algorithm can be adopted in different sustaining mechanisms of AF.

Another observation is that ICAN is very robust to CL change and poor catheter contact (Fig. 6), which makes it suitable for use in clinical settings. The performance of ICAN on simulations with a CL value of approximately 150 and 240 ms demonstrated the algorithm’s robustness to changes of CL in the range of well below and above the baseline CL (178 ± 55 ms) of clinical atrial fibrillation.^20^ The electrodes with poor contact usually are removed from the analysis as they are contaminated by noise and far-field effect and do not represent the local activations.^9^ Our analysis indicated a drop in the algorithm’s performance as more electrodes were missing. However, the algorithm performed relatively well even with 15% of missing electrodes, which translates to at least 11 missing electrodes over a guidance path with seven catheter placements. Unlike spherical basket catheters that cannot establish a contact with a significantly non-spherical atrium, 20-electrode diagnostic catheters are less likely to exhibit poor contact. Along with recent technologies on contact force sensing catheters,^19^ they display good local contact with the atrial tissue.

The localization accuracy of ICAN to detect rotors did not make significant changes with fibrosis-related electrogram changes, such as fractionation and reduced amplitude, made the task of electrogram analysis and source detection in the presence of patchy myocardial scar more challenging. The presence of a far scar from the rotor source or rotor anchoring to a scar area did not affect the performance (Fig. 7a). However, the type of source was incorrectly identified as a focal source in 26.65% of the detected sources in case of a rotor anchoring to a larger area of the myocardial scar. This percentage was reduced to 7.44% after the arrhythmia CL was increased to 240 ms. This suggests that the incorrect identification of the source type is because of the rapid propagation of waves along the Lasso bipoles. These patterns appear as a focal source with simultaneous activation as confirmed by the recorded electrograms at these sites (Fig. 7c, P_1_). For focal sources, the performance was slightly affected by the presence of slow conductions that were formed around the low-voltage area and the resulting electrograms that appeared as a rotational activity along the catheter (Fig. 7c, P_2_). The detection of such slow conductions as a rotor source can be avoided by first applying ICAN to detect the rotor and focal sources separately. Then, the ablation targets can be identified by investigating the location and type of the detected sources as well as the constructed voltage map. Note that the importance of identifying the correct source type depends on the utilized ablation strategy. In ablation strategies where ablation is performed on the core of the detected source,^22^ ICAN can successfully provide the accurate location of the source as the ablation targets.

### Comparison of ICAN with Algorithms Based on Basket Catheter

ICAN is less likely to produce false detections and provides significantly higher accuracy. The phase and divergence maps obtained from the fibrotic tissues using the basket catheter could be very sensitive to catheter orientation and could significantly affect the quality of predictions (Fig. 8). These observations were in agreement with the previous reports^26^ that the low-resolution basket catheters similar to FirMap were prone to false detections. This is a consequence of anisotropic propagation in fibrotic tissues and inherent non-uniformity of electrode density in basket catheters. By their design, individual electrodes in a basket catheter are evenly distributed along eight equally spaced meridional arches. As a result, near the poles, the distance between electrodes in adjacent arches is significantly shorter than the distance between adjacent electrodes within the same arch. Indeed, the rate of false detections is higher, and the accuracy of localization is lower when the meridian of the basket catheter with lower electrode density coincides with the direction of slow propagation. In a Lasso catheter, the individual electrodes are uniformly distributed along the circle. Thus, the catheter orientation plays no role in the performance of ICAN.

### Limitations and Future Work

The ICAN algorithm we developed has the potential to locate patient-specific ablation targets within the atria using a conventional diagnostic catheter with no need of specialized catheters (such as basket catheters) or a laborious process to construct a 3D electro-anatomic map. Our in silico studies indicated a significant potential of ICAN in detecting AF sources with stable sources and atrial tachycardias. Although in silico studies cannot completely substitute for *in vivo* experiments, in silico learning can certainly increase the efficiency of ablation procedures. Performance of ICAN in the presence of AF patterns with very complex and non-stationary activation patterns warrants further investigation.

### Conclusions

We have presented the first algorithm to localize AF sources by iteratively navigating a 20-electrode circular catheter that is routinely used during the AF ablation procedures. The success of the algorithm was verified for accurate detection of source location and type using simulated human atrial model data with 12 electrical remodeling cases to represent complex propagation patterns of AF. This algorithm could enable current clinical electrophysiology laboratory system to detect non-PV AF sources without the need for time-consuming mapping of the entire atria in advance or any assumption on the sustaining mechanism of AF. Such a system may significantly improve the success of patient-specific AF ablation and facilitate treating patients before progression of AF to the permanent stage.

## Electronic supplementary material

Below is the link to the electronic supplementary material.

Supplementary material 1 (PDF 48 kb)

## Abbreviations

ICAN: Iterative catheter navigation
CDR: Conduction delay ratio
PWD: Principal wave direction
ARP: Anatomic re-entry pattern
WDV: Wave divergence

## References

[1] Atienza F, Almendral J, Jalife J, Zlochiver S, Ploutz-Snyder R, Torrecilla E, Arenal A, Kalifa J, Fernández-Avilés F, Berenfeld O (2009). Real-time dominant frequency mapping and ablation of dominant frequency sites in atrial fibrillation with left-to-right frequency gradients predicts long-term maintenance of sinus rhythm. Heart Rhythm.

[2] Atienza F, Almendral J, Ormaetxe J, Moya Á, Martínez-Alday J, Hernández-Madrid A, Castellanos E, Arribas F, Arias M, Tercedor L, Peinado R (2014). Comparison of radiofrequency catheter ablation of drivers and circumferential pulmonary vein isolation in atrial fibrillation: a noninferiority randomized multicenter RADAR-AF trial. JACC.

[3] Benjamin E, Virani S, Callaway C, Chamberlain A, Chang A, Cheng S, Chiuve S, Cushman M, Delling F, Deo R, de Ferranti S (2018). Heart disease and stroke statistics—2018 update: a report from the American Heart Association. Circulation.

[4] Buch E, Share M, Tung R, Benharash P, Sharma P, Koneru J, Mandapati R, Ellenbogen K, Shivkumar K (2016). Long-term clinical outcomes of focal impulse and rotor modulation for treatment of atrial fibrillation: a multicenter experience. Heart Rhythm.

[5] Cherry E, Evans S (2008). Properties of two human atrial cell models in tissue: restitution, memory, propagation, and reentry. J. Theor. Biol..

[6] Cirugeda-Roldán E, Novak D, Kremen V, Cuesta-Frau D, Keller M, Luik A, Srutova M (2015). Characterization of complex fractionated atrial electrograms by sample entropy: an international multi-center study. Entropy.

[7] Frontera A, Takigawa M, Martin R, Thompson N, Cheniti G, Massoullié G, Duchateau J, Wielandts J, Teijeira E, Kitamura T, Wolf M (2018). Electrogram signature of specific activation patterns: analysis of atrial tachycardias at high-density endocardial mapping. Heart Rhythm.

[8] Gharaviri A, Verheule S, Eckstein J, Potse M, Kuklik P, Kuijpers N, Schotten U (2016). How disruption of endo-epicardial electrical connections enhances endo-epicardial conduction during atrial fibrillation. EP Europace.

[9] Ghoraani B, Dalvi R, Gizurarson S, Das M, Ha A, Suszko A, Krishnan S, Chauhan V (2013). Localized rotational activation in the left atrium during human atrial fibrillation: relationship to complex fractionated atrial electrograms and low-voltage zones. Heart Rhythm.

[10] Gima K, Rudy Y (2002). Ionic current basis of electrocardiographic waveforms: a model study. Circ. Res..

[11] Haissaguerre M, Hocini M, Denis A, Shah A, Komatsu Y, Yamashita S, Daly M, Amraoui S, Zellerhoff S, Picat M, Quotb A (2014). Driver domains in persistent atrial fibrillation. Circulation.

[12] Haissaguerre M, Shah A, Cochet H, Hocini M, Dubois R, Efimov I, Vigmond E, Bernus O, Trayanova N (2016). Intermittent drivers anchoring to structural heterogeneities as a major pathophysiological mechanism of human persistent atrial fibrillation. J. Physiol..

[13] Hansen B, Zhao J, Csepe T, Moore B, Li N, Jayne L, Kalyanasundaram A, Lim P, Bratasz A, Powell K, Simonetti O (2015). Atrial fibrillation driven by micro-anatomic intramural re-entry revealed by simultaneous sub-epicardial and sub-endocardial optical mapping in explanted human hearts. Eur. Heart J..

[14] Hunter R, Liu Y, Lu Y, Wang W, Schilling R (2012). Left atrial wall stress distribution and its relationship to electrophysiologic remodeling in persistent atrial fibrillation clinical perspective. Circ. Arrhythm. Electrophysiol..

[15] Jadidi A, Lehrmann H, Keyl C, Sorrel J, Markstein V, Minners J, Park C, Denis A, Jaïs P, Hocini M, Potocnik C (2016). Ablation of persistent atrial fibrillation targeting low-voltage areas with selective activation characteristics. Circ. Arrhythm. Electrophysiol..

[16] Jalife J, Berenfeld O, Mansour M (2002). Mother rotors and fibrillatory conduction: a mechanism of atrial fibrillation. Cardiovasc. Res..

[17] Konings K, Smeets J, Penn O, Wellens H, Allessie M (1997). Configuration of unipolar atrial electrograms during electrically induced atrial fibrillation in humans. Circulation.

[18] Kuklik P, Zeemering S, Maesen B, Maessen J, Crijns H, Verheule S, Ganesan A, Schotten U (2015). Reconstruction of instantaneous phase of unipolar atrial contact electrogram using a concept of sinusoidal recomposition and Hilbert transform. IEEE TBME.

[19] Marijon E, Fazaa S, Narayanan K, Guy-Moyat B, Bouzeman A, Providencia R, Treguer F, Combes N, Bortone A, Boveda S, Combes S (2014). Real-time contact force sensing for pulmonary vein isolation in the setting of paroxysmal atrial fibrillation: procedural and 1-year results. JCE.

[20] Meo M, Pambrun T, Derval N, Dumas-Pomier C, Puyo S, Duchâteau J, Jaïs P, Hocini M, Haïssaguerre M, Dubois R (2018). Noninvasive assessment of atrial fibrillation complexity in relation to ablation characteristics and outcome. Front. Physiol..

[21] Nademanee K, McKenzie J, Kosar E, Schwab M, Sunsaneewitayakul B, Vasavakul T, Khunnawat C, Ngarmukos T (2004). A new approach for catheter ablation of atrial fibrillation: mapping of the electrophysiologic substrate. JACC.

[22] Narayan S, Krummen DE, Rappel WJ (2012). Clinical mapping approach to diagnose electrical rotors and focal impulse sources for human atrial fibrillation. JCE.

[23] Nygren A, Fiset C, Firek L, Clark J, Lindblad D, Clark R, Giles W (1998). Mathematical model of an adult human atrial cell the role of k+ currents in repolarization. Circ. Res..

[24] Pertsove A, Jalife J (1995). Three-dimensional vortex-like reentry. Cardiac electrophysiology: from cell to bedside.

[25] Ravelli F, Faes L, Sandrini L, Gaita F, Antolini R, Scaglione M, Nollo G (2005). Wave similarity mapping shows the spatiotemporal distribution of fibrillatory wave complexity in the human right atrium during paroxysmal and chronic atrial fibrillation. JCE.

[26] Roney C, Cantwell C, Bayer J, Qureshi N, Lim P, Tweedy J, Kanagaratnam P, Peters N, Vigmond E, Ng F (2017). Spatial resolution requirements for accurate identification of drivers of atrial fibrillation. Circ. Arrhythm. Electrophysiol..

[27] Roney C, Cantwell C, Qureshi N, Chowdhury R, Dupont E, Lim P, Vigmond E, Tweedy J, Ng F, Peters N (2017). Rotor tracking using phase of electrograms recorded during atrial fibrillation. Ann. Biomed. Eng..

[28] Shillieto, K., P. Ganesan, A. Salmin, E. Cherry, A. Pertsov, and B. Ghoraani, Catheter simulator software tool to generate electrograms of any multi-polar diagnostic catheter from 3D atrial tissue, *IEEE EMBC*, pp. 2741–2744, 2016. 10.1109/EMBC.2016.7591297 PMC5884094 28268886

[29] Sibson R (1981). Interpolating multivariate data: chapter 2: a brief description of natural neighbor interpolation.

[30] Takahashi Y, O’Neill M, Hocini M, Dubois R, Matsuo S, Knecht S, Mahapatra S, Lim K, Jaïs P, Jonsson A, Sacher F (2008). Characterization of electrograms associated with termination of chronic atrial fibrillation by catheter ablation. JACC.

[31] Wong K, Paisey J, Sopher M, Balasubramaniam R, Jones M, Qureshi N, Hayes C, Ginks M, Rajappan K, Bashir Y, Betts T (2015). No benefit of complex fractionated atrial electrogram (CFAE) ablation in addition to circumferential pulmonary vein ablation and linear ablation: BOCA study. Circ. Arrhythm. Electrophysiol..

