# Supplementary material for: Locating Atrial Fibrillation Rotor and Focal Sources Using Iterative Navigation of Multipole Diagnostic Catheters
Source: Cardiovasc Eng Technol. 2019 Apr 15;10(2):354–66. doi: 10.1007/s13239-019-00414-5 (PMC6527788; doi:10.1007/s13239-019-00414-5)
Supplement: Supplementary file 1 — Supplementary material 1 (PDF 48 kb) [file 13239_2019_414_MOESM1_ESM.pdf]

Supplementary Online Content for “Locating Atrial Fibrillation Rotor and Focal Sources Using Iterative Navigation of Multipole Diagnostic Catheters”

Table of Contents:

1. **AF Source Initiation**
2. **Basket Catheter Simulation**
3. **Detecting AF sources using basket catheter**
4. **References**

1. AF Simulation Methods

2D simulation: The simulations were carried out on a 10cm x10cm with a spatial resolution of 0.25 mm. To model tissue fibrosis, collagenous septa were introduced by removing electrical connections between neighboring myocytes oriented along the assumed fiber orientation (left-to-right) and uniformly distributed throughout the sample[S6]. The septa lengths varied according to a Poisson distribution[S6] with an average length of 2.5mm[S3] and disrupted lateral coupling in 20% of the tissue[S1]. We simulated AF with different cycle length (CL) values by increasing the K+ conductance value to shorten the cycle length, and increasing the Na+ conductance value to generate longer cycle lengths[5]. In some simulations (**Figure 2**-E-I and K), in addition to fibrosis we also introduced patchy myocardial scars corresponding to “low voltage” areas, which are common for pathological atrial myocardium[12]. Patchy myocardial scars were generated by randomly removing 50% of electrical connections within a localized square area. To simulate fibrillatory activation patterns with non-sustained singularity point rotors[16], in some simulations (**Figure 2**-L) we also introduced spatial heterogeneity in inward rectifier channel conductance by increasing the I_{K1} conductance, which has been shown to be linked to AF related remodeling[S2] in the lower half of the simulated area.

3D simulation: The simulations were performed on a real 3D anatomy based on the Harrild and Henriquez model[S4] including: the left atrium, right atrium, Bachmann's bundle, pectinate muscles, left atrial appendage, fossa ovalis, and the superior and inferior vena cava. The mean element size of the model is 550μm and the maximum size is 1,650μm. Collagenous septa were generated by removing the electrical connections in the transmural direction perpendicular to the myocardial fiber orientation. The septa was created in the form of 2.5mmx2.5mm blocks at 400 randomly sampled (uniformly distributed) locations, with the length of septa sampled from a Poisson distribution with average length of 2.5mm. The 3D left atrium was segmented from the complete 3D anatomy and the data was used to test our algorithm.

AF Source Initiation: We generated several test cases with AF rotor and focal sources in 2D and 3D simulations. In all cases, rotors were initiated by a cross-field stimulation protocol[5] and focal sources were initiated by providing a stimulus with an amplitude of

20mV and area of 1.25x1.25mm. To simulate functional figure-of-eight re-entry, we simulated two closed spaced rotor cores. **Figure 2** shows 12 of the simulations.

2. Basket Catheter Simulation

To compare the performance of the ICAN algorithm with the phase-mapping approach we simulated a 64-electrode FirMap atrial basket catheter comprising of 8 splines with 8 evenly spaced electrodes in each spline. We simulated a basket catheter of 40mm in diameter (the highest resolution among the existing FirMap basket catheters) with an in-between electrode spacing of 6.8 mm in each splines that has been scaled accordingly to the resolution of our 3D anatomy.

In the case of 2D atrial tissue, the catheter was mapped onto the endocardial surface as a rectangular electrode array with spacing 15.7mm x 6.8mm (**Figure 3B**). A total of 120 simulations of 2D catheter placement was generated by shifting and rotating the basket catheter on the 2D atrial tissue. Three shifts (spacing of 1cm) were performed in the highest resolution direction and 8 shifts (spacing of 1cm) were performed in the lowest resolution direction. For every basket simulation, five different orientations (0^0, 30^0, 45^0, 60^0, and 90^0) were simulated.

The 3D simulation of the basket catheter was performed considering the realistic non-uniformity of electrode density in basket catheters. The 8 electrodes on one spline were evenly placed according to Eq. (2).

$$u(n) = \frac{r}{\Delta h}\begin{bmatrix}\cos(\theta_u n + \phi)\\ \sin(\theta_u n + \phi)\end{bmatrix} + \begin{bmatrix}x_c\\ y_c\end{bmatrix} \quad (2)$$

where $u(n)$ indicates the coordinates of $n = \{1, \ldots, 8\}$ unipole electrodes on one spinals of a catheter centered at (x_c, y_c) , $r = 20mm$ is the catheter radius, $\Delta h = 0.25$ mm is the spatial resolution, $\theta_u = 19.5°$ is the spacing between unipoles, and $\phi = 21.8°$ is to account for the distance between electrodes 1 and 8 from the beginning and ending of the spinal. The spline was then rotated 7 times with an azimuth of $45°$ to generate the coordinates of the electrodes on the remaining splines. A total of three simulations of 3D catheter placement was generated by rotating the basket catheter with an azimuth of $11.25°$.

3. Detecting AF sources using basket catheter

We performed the following analysis to detect rotor or focal sources using a 64-electrode FirMap atrial basket catheter. First, the extracellular voltage from the unipolar electrograms was spatially interpolated using bilinear interpolation to achieve a grid spacing of at least 4mm in all directions.

Phase mapping to detect rotors: The phase map was calculated between $\pm\pi$ using the phase-mapping algorithm by Kuklik *et. al*[18]. In 2D atrial tissue, the phase singularity points were identified from the generated phase maps by applying the Iyer-Gray algorithm[S5]. In 3D atrial tissue, we used an approach based on method in Ref. [S7] to

detect phase singularities. Finally, clusters of phase singularity points with less than 4mm distance from the center of the cluster were identified and those lasting greater than one wave propagation cycle were considered to be rotors[27].

Velocity-of-divergence mapping to detect focal sources: We followed an approach similar to the method described in Ref. [27]. For every four neighboring unipolar recordings, an average conduction velocity vector was determined by averaging the isochrone vectors (same as in WDV calculation) obtained from the activation times of the three-recording combinations. Next, the divergence field of all the conduction velocity vectors was computed and the locations with divergence values of greater than the 95th percentile of the values in the field and those lasting more than one wave propagation cycle were identified as focal sources.

[S1] Akoum, N., M. Daccarett, C. McGann, N. Segerson, G. Vergara, S. Kuppahally, T. Badger, N. Burgon, T. Haslam, E. Kholmovski and R. Macleod, "Atrial fibrosis helps select the appropriate patient and strategy in catheter ablation of atrial fibrillation: a DE-MRI guided approach," *JCE,* 22(1):16-22, 2011.

[S2] Berenfeld, O., "The major role of I K1 in mechanisms of rotor drift in the atria: a computational study," Clinical Medicine Insights: Cardiology, 10:CMC-S39773, 2016.

[S3] Dolber, P. and M. Spach, "Thin collagenous septa in cardiac muscle," *Anatomical Record,* 218(1):45-55, 1987.

[S4] Harrild, D. and C. Henriquez, "A finite volume model of cardiac propagation," *Ann Biomed Eng,* 25(2):315-334, 1997.

[S5] Iyer, A. and R. Gray, "An experimentalist's approach to accurate localization of phase singularities during reentry," *Ann Biomed Eng,* 29(1): 47-59, 2001.

[S6] Jacquemet, V. and C. Henriquez, "Genesis of complex fractionated atrial electrograms in zones of slow conduction: a computer model of microfibrosis," Heart Rhythm, 6(6):803–810, 2009.

[S7] Rogers, J., "Combined phase singularity and wavefront analysis for optical maps of ventricular fibrillation," *IEEE TBME,* 51(1): 56-65, 2004.
